# An interleukin 6 responsive plasma cell signature is associated with disease progression in systemic sclerosis interstitial lung disease

**DOI:** 10.1016/j.isci.2023.108133

**Published:** 2023-10-05

**Authors:** Guiquan Jia, Thirumalai R. Ramalingam, Jason Vander Heiden, Xia Gao, Daryle DePianto, Katrina B. Morshead, Zora Modrusan, Nandhini Ramamoorthi, Paul Wolters, Celia Lin, Dinesh Khanna, Joseph R. Arron

**Affiliations:** 1 Genentech Inc, South San Francisco, CA 94080, USA; 2 UCSF, San Francisco, CA 94143, USA; 3 University of Michigan, Ann Arbor, MI 48104, USA

**Keywords:** Fibrosis, Biological sciences, Molecular biology, Immunology

## Abstract

Systemic sclerosis (SSc) interstitial lung disease (ILD) is among the leading causes of SSc-related morbidity and mortality. Tocilizumab (TCZ, anti-IL6RA) has demonstrated a reduced rate of pulmonary function decline in two phase 2/3 trials (faSScinate and focuSSced) in SSc-ILD patients. We performed transcriptome analysis of skin biopsy samples collected in the studies to decipher gene networks that were potentially associated with clinical responses to TCZ treatment. One module correlated with disease progression showed pharmacodynamic changes with TCZ treatment, and was characterized by plasma cell (PC) genes. PC signature gene expression levels were also significantly increased in both fibrotic SSc and IPF lungs compared to controls. scRNAseq analyses confirmed that PC signature genes were co-expressed in CD38 and CD138 expressing PC subsets in SSc lungs. These data provide insights into the potential role of PC in disease progression and mechanisms of action of TCZ in fibrotic interstitial lung diseases.

## Highlights

• Tocilizumab treatment benefits SSc-ILD patients with stabilization of lung function
• Skin plasma cell (PC) gene signature is identified responsive to the treatment
• The enriched PC signature is associated with lung function of SSc-ILD patients
• The enriched PC signature is interrogated and verified in the lung of ILD patients

## Graphical abstract

**Figure:**
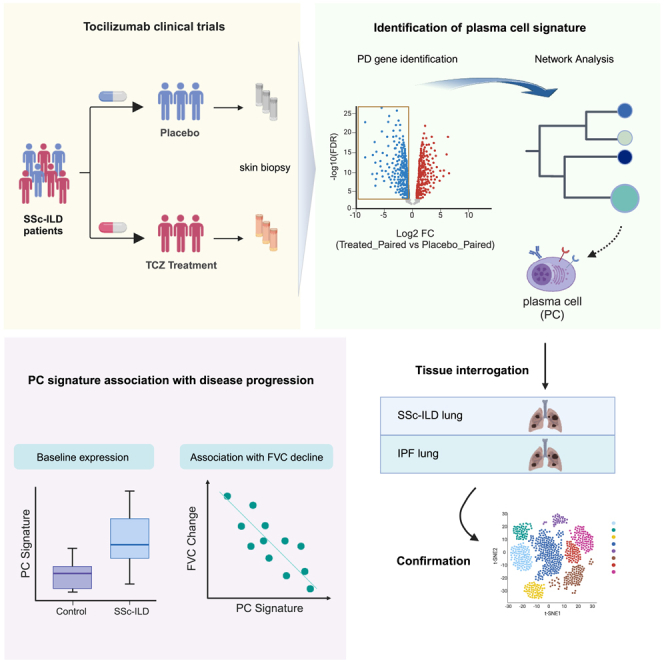

## Introduction

Systemic sclerosis (SSc) is an autoimmune disease characterized by fibrosis, vascular dysfunction, and immune dysregulation. SSc often initially presents as Raynaud’s phenomenon, a manifestation of microvasculopathy, with patients testing positive for one or more of a well-defined suite of autoantibodies.^1^ While the exact pathogenesis of SSc remains poorly understood, aberrant activation of immune cells abetted by autoantibodies and immune complexes can trigger repeated bouts of tissue injury and activate wound healing pathways that manifest over time as organ fibrosis. While a variable degree of skin involvement is almost universally present in SSc patients, significant morbidity arises from involvement of internal organs such as lungs, kidneys, and gastrointestinal tract. Indeed, interstitial lung disease (ILD) stands among the leading causes of death in SSc patients^2^^,^^3^ and affects nearly 80% of SSc patients over their lifetimes. Though diffuse skin involvement and certain autoantibody repertoires are associated with greater risk of disease progression, there are no validated biomarkers shown to be clinically useful in prognosticating disease trajectory, or predicting treatment response.

Interleukin 6 (IL-6) has been postulated to be involved in various aspects of SSc pathogenesis, including both activation and recruitment of myeloid and B cells to the lesions.^4^^,^^5^^,^^6^ Tocilizumab (TCZ), a monoclonal antibody against IL-6RA that inhibits IL-6 activity,^7^ was recently tested in SSc patients in two large phase 2/3 trials.^8^^,^^9^ Although TCZ treatment did not significantly improve skin thickening measured by modified Rodnan skin score (MRSS),^10^ lung function, measured as change in forced vital capacity (FVC) over time, was significantly stabilized in patients treated with TCZ. A key question is whether the lack of significant benefit on the clinical MRSS outcome is reflected in pharmacodynamic effects in skin tissue. An objective assessment of changes in disease pathway activity via molecular analysis may be more relevant and robust in quantifying the mechanistic contribution of the targeted biology to SSc pathogenesis.

SSc lends itself to molecular characterization as lesional skin punch biopsies can be readily collected before and after treatment. Assessment of disease activity at the molecular level, using transcriptomic and/or proteomic approaches, can enable us to explore prognostic relationships between activity levels of various pathways to key clinical manifestations of the disease and their progression over time, pharmacodynamics of targeted pathways in response to treatment, and potentially explore predictive aspects of certain molecular signatures that enrich for clinical benefit with a given intervention. Such molecular approaches are instrumental to realizing the promise of stratified medicine such that the right patient receives the right drug at the right time in the disease course. Multiple studies have reported on the heterogeneity of skin gene signatures in patients with SSc,^11^^,^^12^^,^^13^ and some have correlated these signatures with cross-sectional clinical metrics, but very few have done so in a large, well curated cohort of patients followed up systematically for an extended period of time to allow for the assessment of disease progression. In order to understand IL-6 modulated cellular and molecular signals dysregulated in SSc patient skin, we performed unbiased RNAseq analyses of skin punch biopsies collected as part of a large phase 3 study of TCZ in SSc, and identified a gene signature attributable to plasma cells (PCs) that is elevated in SSc skin and correlated with disease progression. This signature was blunted by TCZ treatment, thereby implicating a role for IL-6 in plasma cell (PC) mediated pathobiological processes in SSc.

## Results

### Demographic profile of the subjects in the study

A total 212 subjects with diffuse cutaneous SSc were enrolled in the focuSSced study and randomly assigned to receive placebo or tocilizumab (TCZ) as detailed elsewhere.^8^ 208 subjects with non-missing baseline and follow up clinical data were used in the post-hoc study. Among them, skin RNA samples were available from 37 subjects that consented for an optional, exploratory biopsy, collected once immediately prior to treatment (baseline, BL), and followed by a second biopsy at 48 weeks of treatment—including 18 from the active treatment arm and 18 from the placebo arm (of which 11 subjects in each arm had one biopsy at each time point) after the removal of outlier samples from RNAseq (see STAR Methods section for detail). Baseline demographics and disease characteristics for the subjects in the entire study and the subset with skin biopsy samples used in this analysis are presented in Table 1.

**Table 1:** Characterization of demographics of subjects in comparison between RNAseq subset and the total cohort of FocuSSced

Comparison of population characteristics between subjects in the total cohort and RNAseq subset			
	RNAseq subset	Total cohort	p value
N	37	208	
Age (mean (SD))	51.27 (11.25)	48.09 (12.43)	n.s.
FVC%pred. (median [IQR])	81.35 [70.19, 93.44]	82.48 [71.12, 93.30]	n.s.
FVC change (median [IQR])	−0.05 [-0.16, 0.06]	−0.05 [-0.12, 0.06]	n.s.
DLCO%pred. (median [IQR])	74.29 [57.28, 89.30]	73.63 [63.21, 88.28]	n.s.
DLCO change (median [IQR])	−0.09 [-0.23, 0.01]	−0.05 [-0.17, 0.09]	n.s.
MRSS (median [IQR])	22.00 [17.00, 30.00]	19.00 [15.00, 25.00]	∗
MRSS change (median [IQR])	−0.11 [-0.20, −0.01]	−0.12 [-0.20, −0.02]	n.s.

Comparison of population characteristics within PBO arm			
	RNAseq subset	Total cohort	p value
n	19	104	
Age (mean (SD))	54.05 (10.74)	49.19 (12.66)	n.s.
FVC%pred. (median [IQR])	92.13 [81.48, 98.03]	86.84 [72.56, 95.96]	n.s.
FVC change (median [IQR])	−0.13 [-0.28, −0.03]	−0.08 [-0.16, 0.01]	n.s.
DLCO%pred. (median [IQR])	76.55 [69.66, 98.06]	76.15 [65.70, 85.90]	n.s.
DLCO change (median [IQR])	−0.14 [-0.31, −0.03]	−0.06 [-0.18, 0.07]	n.s.
MRSS (median [IQR])	20.00 [15.50, 29.00]	19.00 [15.00, 26.00]	n.s.
MRSS change (median [IQR])	−0.05 [-0.19, 0.02]	−0.11 [-0.20, 0.01]	n.s.

Comparison of population characteristics within TCZ arm			
	RNAseq subset	Total cohort	p value
n	18	104	
Age (mean (SD))	48.33 (11.31)	46.98 (12.15)	n.s.
FVC%pred. (median [IQR])	72.89 [66.23, 77.70]	80.00 [69.69, 90.04]	n.s.
FVC change (median [IQR])	0.01 [-0.08, 0.18]	−0.02 [-0.10, 0.08]	n.s.
DLCO%pred. (median [IQR])	60.85 [51.15, 82.33]	71.46 [59.42, 89.24]	n.s.
DLCO change (median [IQR])	−0.04 [-0.12, 0.11]	−0.05 [-0.17, 0.11]	n.s.
MRSS (median [IQR])	25.50 [19.00, 30.75]	19.00 [15.00, 24.25]	∗∗
MRSS change (median [IQR])	−0.18 [-0.23, −0.06]	−0.14 [-0.21, −0.06]	n.s.

n.s.: no significance statistically; ∗∗ p value <0.05.

The overall demographic profile was generally comparable between the biopsy subset and the total cohort, including age at baseline, gender distribution, baseline lung function as measured by percent predicted FVC (median FVC%pred.: 81.35 in the subset vs. 82.48 in the total cohort) and percent predicted diffusing capacity of lung for carbon monoxide (median DLCO%pred.: 74.29 in the subset, vs. 73.63 in the total). However, degree of skin involvement at baseline, as measured by the MRSS was higher in the biopsy subset than the total cohort (median MRSS: 22.0 vs. 19.0, p value <0.05). The key clinical endpoints, including change from baseline to week 48 in MRSS and in lung function, as measured by linear regression slope using all longitudinal measurements, were not meaningfully different between the subset and the entire cohort.

### Identification of a distinct plasma cell (PC) gene module from pharmacodynamic effects of TCZ treatment on skin transcriptional profiles

**Figure 1:**
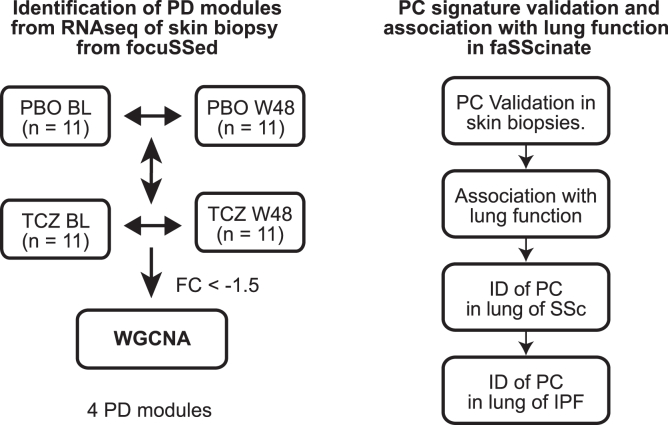
Flow chart of the data analysis The figure summarizes the main procedures and steps in the entire data analysis; the left panel illustrates the analytical steps for the identification (ID) of pharmacodynamic (PD) modules, while the right panel shows the validation of plasma cell (PC) as PD module associated with the disease. BL: baseline; W48: week 48; FaSS: FaSScinate; PC: plasma cells; PD: pharmacodynamic; PBO: placebo group; TCZ: the group of treatment with tocilizumab; WGCNA: weighted gene co-expression network analysis.

**Figure 2:**
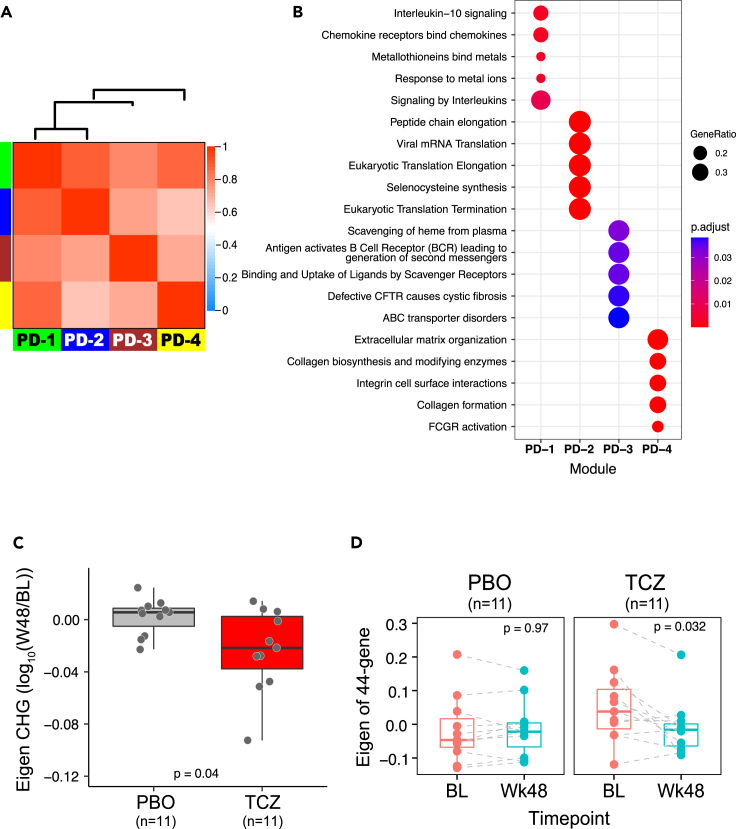
Identified PD modules from RNAseq of skin biopsy (A) The Module eigengene adjacency dendrogram (top) and heatmap with red and blue indicating highly related and unrelated modules, respectively, depicting the relationship of the modules of PD genes identified by WGCNA. The color row below the heatmap indicates PD module assignment: PD-1 (n = 33 genes), PD-2 (n = 211 genes), PD-3 (n = 44 genes), and PD-4 (n = 44 genes). (B) Reactome pathway enrichment of each module gene set. Pathways were determined for each module gene using the compareCluster function with Reactome database. The top 5 of the most over-represented the classification of pathways are illustrated as dot plots, with the gene ratio denoted by size and the significance denoted by color. The p values were adjusted by the Benjamini-Hochberg method. (C) PC module eigengene (ME) was down-regulated in TCZ treatment group compared with placebo group in the FocuSSced cohort. The eigengene change of PC module 48 weeks post treatment from pretreatment is expressed as log10 on y axis; the p value (Wilcoxon test) is indicated at the bottom of graph. (D) Paired comparison of module eigengene at baseline and 48 weeks following-up. Intra-patient comparison showed a significant reduction of ME in patients with TCZ treatment but not with placebo (PBO).

We performed RNAseq on bulk RNA extracted from available skin biopsies and queried whether any genes/pathways pharmacodynamically (PD) responded to TCZ treatment. We performed differential gene expression analysis in a design of time course with nested individuals for group-specific effects, where we compared baseline to week 48 expression changes intra-individually for individuals with both pre- and post-treatment biopsies prior to aggregating those changes for each group and then compared the aggregate changes between TCZ treatment and the placebo (PBO) groups. Overall, 534 genes from 764 DEG (defined as differentially expressed genes (DEG) with p value <0.05 & |fold change| > 1.5, as detailed in the Methods section) were significantly down-regulated in the TCZ treatment group compared to PBO over time. These TCZ down-regulated genes were advanced as a candidate PD gene set. To identify molecular pathways implicated by these genes, we used weighted gene co-expression network analysis (WGCNA) across all PD genes to construct co-expressed networks and module partitions (see STAR Methods section and Figure 1 for details). These analyses identified a total of four modules (designated as PD-1 to PD- 4) as shown in Figure 2A (detailed gene list in each module in supplemental information). We performed pathway enrichment analysis to identify biological pathways that were implicated by the genes in the modules and top 5 enriched pathways are listed in the dotplot (Figure 2B). The module PD-1 (including 33 genes) was enriched for pathways involved in the regulation of immune responses; module PD-2 (211 genes) was relatively proximal to PD-1 in correlation and comprised many genes involved in the pathways and cellular functions responsible for gene translation elongation and termination. The genes in the module PD-4 (44 genes) were involved in pathways related to wound healing processes, such as extracellular matrix organization, collagen biosynthesis, and macrophage activation, e.g., Fc-gamma receptor (FCGR) activation (Figure 2B). Some genes in PD-4 have shown significant reduction in expression as a macrophage gene set in response to TCZ treatment in the FaSScinate cohort,^14^ including FCGR1A, FCGR3A, CD163L, C1QB, NNMT, and VSIG4.

Interestingly, the PD-3 module comprising 44 genes (gene set list in supplemental information) was highly enriched for transcripts associated with PCs (PC, also termed as antibody secreting cells), most of which were immunoglobulin variable region genes (39 out of 44) and others implicated in PC function and/or known to be selectively expressed in PC (MZB1, POU2AF1, DERL3, FAM30A, and CD79A). For instance, the gene MZB1, encoding marginal zone B and B1 cell specific protein, is required for T cell-independent immune responses and differentiation of PCs^15^ and POU2AF1 is a B cell-specific transcriptional coactivator.^16^^,^^17^ Therefore, we designated this gene module the PC module. PC module expression, represented by module eigengene (ME), was significantly reduced following TCZ treatment (Figure 2C). The heterogeneity in the degree of PD response of the PC module may in turn be related to the overall heterogeneity of the underlying disease among SSc subjects. Subjects showing the greatest PD effect of TCZ treatment generally tended to be those with higher baseline PC module expression within the group, whereas there was no significant change over time in this expression module in the placebo group (Figure 2D; Figure S1).

Immunoglobulin (Ig) variable region genes are uniquely expressed in B-lineage cells and at very high levels in Ig-secreting cells such as plasmablasts and PCs, hence their presence in the PD-3 module specifically implicates Ig-secreting cells as a major component of this module. However, Ig variable region genes are not always well annotated or individually informative in gene expression datasets, due to their intrinsic complicated sequence profiling based on antigen dependency across individuals. Therefore, we used the first four non-immunoglobulin genes (MZB1, POU2AF1, DERL3, and FAM30A) in the module that we also found highly correlated in lungs later as an abbreviated PC gene signature in further analyses unless otherwise noted. We tested whether this four-gene signature could faithfully recapitulate overall PC ME and confirmed that it indeed highly and significantly correlated with the full PC module (R = 0.96, p < 0.001; Figure S2A) and showed the same PD effect of TCZ treatment (Figure S2B)

### Validation of PD effect on PC gene signature in an independent cohort

**Figure 3:**
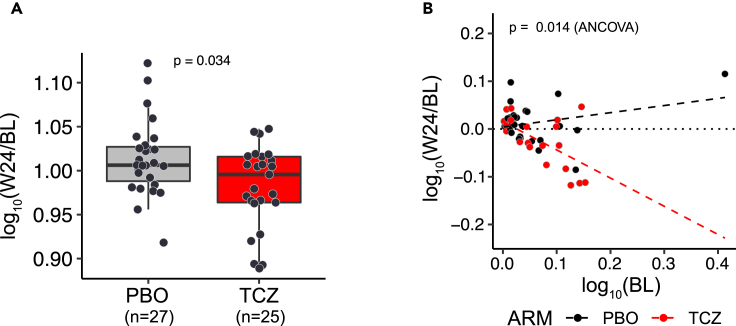
PC signature is down-regulated by TCZ treatment in FaSScinate cohort (A) PC signature is down-regulated in TCZ treatment arm compared with placebo arm. The eigengene change of 3-gene PC signature 24 weeks post treatment from pretreatment is expressed on y axis and p value labeled on the top of graph is from Wilcoxon test. (B) Additional test with ANCOVA model confirmed the significant down-regulation of PC signature by TCZ treatment and the greater PD effects on the subjects with higher PC expression level. Scatterplot shows the function of change (follow-up/baseline measurements) against baseline measurements, both in log10. The dotted line represents perfect agreement (no change) and the dashed lines are fitted regression lines for TCZ treatment and placebo groups. P value was from ANCOVA test.

To validate our observations in an independent cohort, we used skin biopsy transcriptomic data available from the faSScinate study,^14^ which recruited a similar profile of SSc patients and used a clinical trial design analogous to focuSSced, with a few notable differences. The follow-up biopsy was conducted 24 weeks after treatment, compared to 48 weeks in focuSSced, and the transcriptomic data were captured using a whole transcriptome microarray technology, as opposed to the RNA sequencing approach used for focuSSced. Given platform differences, only three of the four genes from the abbreviated PC module (MZB1, POU2AF1, and DERL3) were represented in the microarray dataset, and used as the PC gene set in this case. Consistent with the observations for the four-gene PC module in focuSSced, this three-gene set in FaSScinate showed significant down-regulation following TCZ treatment as compared to placebo control (Figure 3A) and highly correlated each other (Figure S3). Some of the samples in the placebo group that had higher PC eigengene values at baseline showed a spontaneous reduction at 24 weeks (data not shown), suggesting a possible “regression to the mean” (RTM) phenomenon commonly observed with repeated measures in clinical trials,^18^ or a natural change in the underlying disease activity. In order to assess and adjust for the effect of RTM, we performed an analysis of covariance (ANCOVA),^19^ factoring the baseline PC eigengene levels as a covariate (Figure 3B). This analysis showed that TCZ treatment had a statistically significant PD effect compared to the PBO arm. In addition, the divergence between the regression lines by increasing baseline PC gene expression levels confirmed the observation in focuSSced that higher PC expression levels at baseline corresponded to a greater potential to observe a PD effect with TCZ treatment.

### Skin PC module expression is elevated and prognostic of FVC decline in SSc-ILD patients

**Figure 4:**
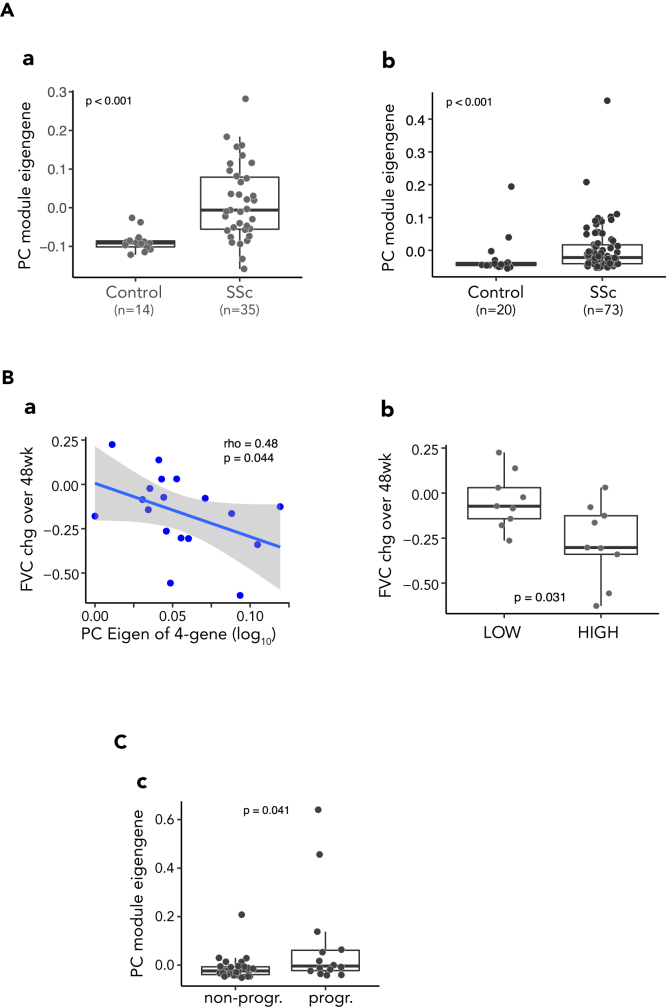
Elevated PC signature was associated with FVC decline in SSc-ILD (A) Baseline PC module is highly elevated in SSc skin compared with healthy controls. (a) PC module eigengene in FocuSSced; (b) 3-gene eigengene in FaSScinate. (B) Baseline PC module expression associated with FVC decline over 48 weeks. (a) PC module eigengene (ME) in skin biopsies was correlated inversely with FVC change (percent predicted) in placebo group of the FocuSSced study. The Spearman correlation coefficient and p value are listed on the top of the plot. (b) Subjects dichotomized according to median baseline ME showed significant differences in FVC change. P value was from Wilcoxon test. LOW: the group with baseline ME less than the median; HIGH: the group with baseline ME greater than the median. (C) The progressors in lung function decline showed higher PC gene expression in the placebo arm of FaSScinate cohort. Progressors (progr.) were defined as subjects with FVC decline over 48 weeks ≥ 10% or DLCO decline >15%. P value was from Wilcoxon test.

The PC gene module showed elevated expression levels in SSc skin samples compared to age and gender matched healthy controls (Figure 4A) in both focuSSced (Figure 4A[a]) and faSScinate (Figure 4A[b]) clinical studies. This is consistent with the observed greater PD effect on the PC module in patients with higher baseline activity of the pathway, as they had a detectable window to show decreases in pathway activity.

As TCZ treatment was associated with a reduced rate of lung function decline in SSc patients in both focuSSced and faSScinate studies, we queried whether skin PC biology and gene expression levels would be informative in the context of SSc lung disease. In the placebo arm of the focuSSced study, where lung function progressively decreased over time, we found that patients with higher level of skin PC module gene expression at baseline showed greater lung disease progression, as measured by FVC change over time (using all the available FVC data from baseline to wk 48) (Figure 4B[a]). Specifically, subjects with higher than median levels of PC signature at baseline showed significantly larger FVC decline over time (Figure 4B[b]).

Although the skin PC eigengene at the baseline did not show significant correlation with continuous change in the FVC over 48 weeks in faSScinate (data not shown) possibly due to the eigengene value calculated from the gene expression derived from the microarray and/or unexplained baseline differences between the subjects in these two clinical trials,^8^^,^^14^^,^^20^ using a combination of categorical definition of disease progression (FVC decrease >10% or DLCO decrease >15% over 48 weeks) which had greater prognostic significance than continuous change in the FVC,^21^ subjects with “progressive ILD” showed higher PC eigengene values at baseline than non-progressors in the faSScinate study (Figure 4C).

Interestingly, baseline PC eigengene level did not show a significant correlation with longitudinal changes in skin thickness (MRSS), an endpoint that TCZ treatment failed to significantly impact in both the clinical trials, although there was a nonsignificant trend for a baseline correlation between PC score and MRSS. Nor was a significant correlation evident between PC eigengene and diffusing capacity of the lungs for carbon monoxide (DLCO), either at baseline level or the change over time (Figure S4).

### Skin PC signature is enriched in lungs of SSc-ILD patients

MZB1 protein and MZB1-positive PC have been reported to be enriched in both human lung and skin fibrosis,^22^ suggesting that PC accumulation as a common underlying pathological phenomenon in both skin and lung of SSc patients.

**Figure 5:**
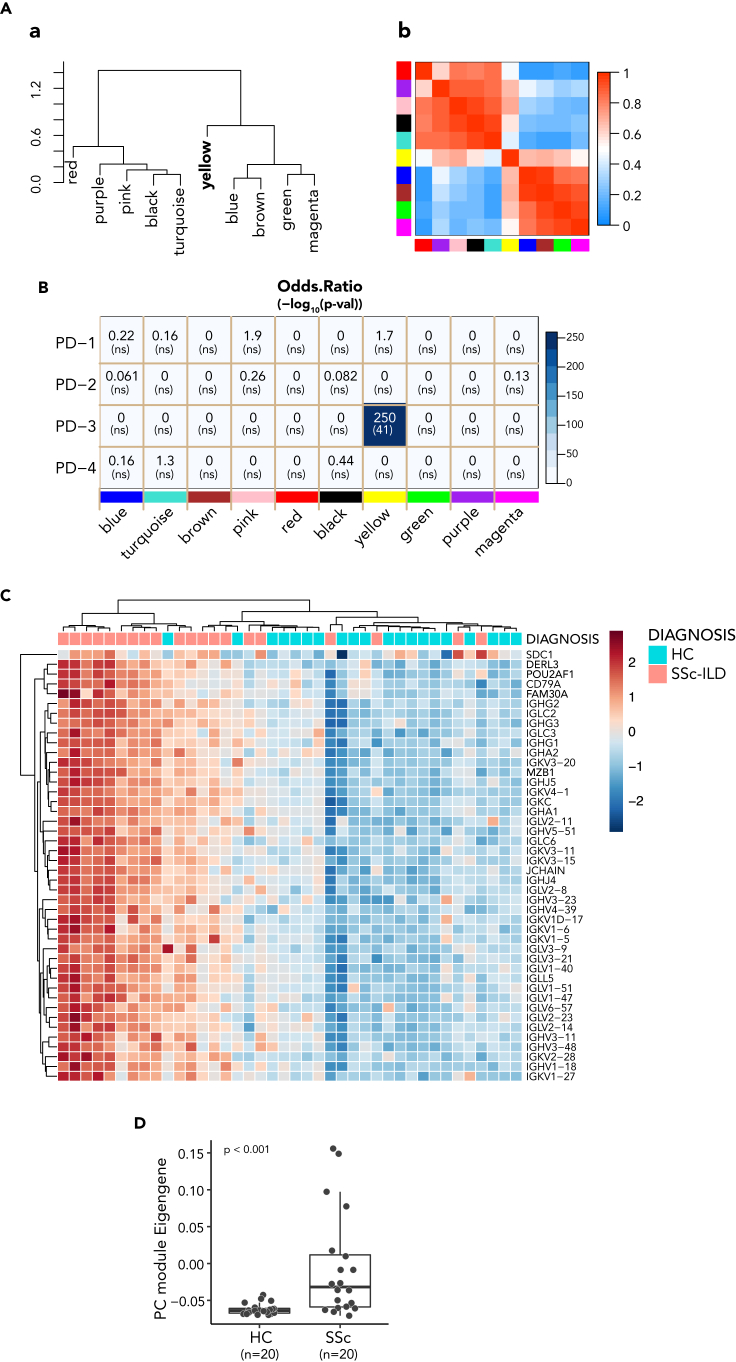
Skin PC signature enriched in SSc-ILD lung (A) Relationship between WGCNA derived module eigengenes from RNAseq of SSc-ILD lung. (a) The eigengene dendrogram of DEG genes on hierarchical clustering of adjacency-based dissimilarity, and (b) relationship of modules on eigengene adjacency heatmap. The modules assigned in WGCNA were either text labeled in (a) or color labeled on both axes of the heatmap in (b), respectively. Colors in heatmap are from low adjacency (blue) to high adjacency (red). (B) Heatmap showing the overall results from overlap analysis between the module genes from SSc-ILD lung and PD modules from SSc skin. Blue color gradient represents the odds ratio score. The number on the top in each square box of heatmap represents the odds ratio value from the analysis, while the number in the parentheses at the bottom represents the p value in -log10. The color labeled modules derived from the RNAseq of SSc-ILD lung are on the x axis and 4 PD modules from skin RNAseq are on y axis. ns represents no statistical significance with a cutoff of p < 0.05. (C) Heatmap of elevated PC gene expression in RNAseq of SSc-IL lungs compared with control lungs. PC module genes (n = 44) in rows and subjects in columns were hierarchically clustered and the gene expression was transformed and standardized to *Z* score. The conventional PC marker CD138 (encoded by SDC1) was included as a reference displayed on the top of row in the heatmap. The four PC signature genes are highlighted in bold in the label. (D) Elevated PC ME in lung of SSc-ILD. PC ME of SSc-ILD lung was compared with controls. p value shown on the top of plot was derived from Wilcoxon test.

To build upon these observations, we generated RNAseq data from explanted SSc-ILD and control lung tissue and determined the DEG between the two groups. We subjected this subset of genes to WGCNA analysis to identify gene co-expression modules, coded by color names (Figure 5A). The modules aggregated into two main groups with network adjacency matrix, while one module (labeled yellow) was less strongly correlated with either of the two main groups.

We performed gene similarity analysis of pathways^23^ to compare the lung co-expression modules to the skin PD modules to assess whether any were related to skin gene sets of interest. The overall results from the overlap analysis are shown in an odds ratio heatmap (Figure 5B). Intriguingly, the yellow module was identified in lung as most overlapping with the skin PC module (PD-3), whereas none of the other lung modules significantly overlapped with any of the four skin PD modules. The yellow module comprised 195 genes, among which 133 genes encode immunoglobulin genes, in addition to many other genes relevant to B cell and PC biology.

We performed a hierarchical clustering of the gene expression levels of the skin PC module (n = 44) in lungs from subjects with SSc and controls (Figure 5C). These genes were coordinately expressed at higher levels in a majority of SSc lungs analyzed, relative to controls. Consistent with the expression of PC module in skin, the skin PC ME was significantly higher in SSc lungs compared to controls (Figure 5D).

It was infeasible given the limitations of the study design to sample lung tissue longitudinally in the context of TCZ treatment to determine whether the PD effect on the PC signature observed in skin is also evident in lung. However, given the evidence that the PC signature is elevated in both fibrotic lung and skin tissue and modulated by TCZ treatment in skin, and that TCZ treatment significantly stabilized lung function in SSc-ILD, it is plausible that increased tissue PC activity might actively contribute to lung disease progression in ILD.

Given the increased PC signature in skin and lung tissues, we explored whether the PC related transcripts were elevated in peripheral blood of SSc patients as well. Analysis of data from RNAseq of whole blood samples collected from the FaSScinate cohort at baseline showed that tissue PC signature genes were not co-expressed in a similar manner in blood as in tissues. Nevertheless, some PC genes (such as DERL3 and POU2AF1) were down-regulated in SSc-ILD blood compared the controls (Figure S5). The tissue PC module we discovered in response to IL6 inhibition may therefore be distinct from that of circulating leukocytes, which may reflect differential recruitment, retention, and/or redistribution of these cells in affected tissues.

### Skin PC signature from SSc-ILD is also enriched in IPF ILD lungs

**Figure 6:**
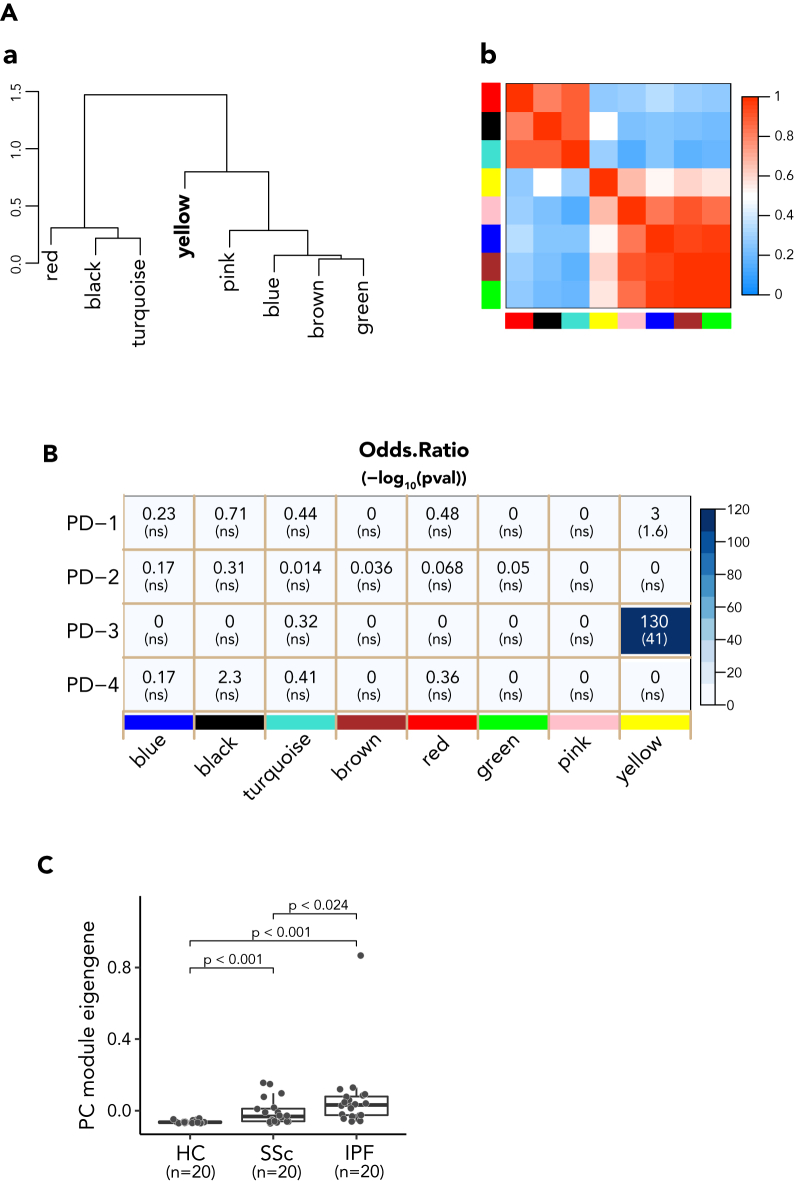
Skin PC signature enriched in IPF ILD lungs (A) Relationship between WGCNA derived module eigengenes from RNAseq of IPF lung. (a) The eigengene dendrogram of DEG genes on hierarchical clustering of adjacency-based dissimilarity, and (b) relationship of modules on eigengene adjacency heatmap depicted tight clusters of correlated eigengenes of modules. The modules assigned in WGCNA, were either text labeled in (a) or color labeled on both axes of the heatmap in (b), respectively. Colors in heatmap are from low adjacency (blue) to high adjacency (red). (B) Heatmap showing the overall results from overlap analysis between the module genes from IPF lung and PD modules from SSc skin. Blue color gradient represents the score of odds ratios. The number on the top in each square box of heatmap represents the odds ratio of overlapping from the analysis, while the number in parentheses at the bottom represents the p value in -log10. The color labeled modules derived from the RNAseq of IPF lung are present on x axis and 4 PD modules from skin RNAseq are on y axis. ns represents no statistical significance with a cutoff of p < 0.05. (C) PC ME in control and ILD lungs. PC MEs of SSc and IPF lungs were compared to each other and to controls (n = 20 in each category as shown). P value shown on the top of plot was derived from Wilcoxon test.

Next we evaluated whether the increased expression of PC module genes was unique to SSc lungs, or if the feature is readily apparent in other fibrotic interstitial lung diseases as well. For this, we compared RNAseq of lung tissue explants from idiopathic pulmonary fibrosis (IPF) patients and that of controls, and analyzed the DEG in IPF patients compared to controls using WGCNA (Figure 6A). Interestingly, akin to SSc-ILD lungs, gene modules in IPF lungs aggregated into two major groups plus one module (yellow) that was less related to either of the major groups. The IPF yellow module was highly similar to the corresponding module in SSc lungs, displaying a large odds ratio and low p value (Figure 6B). This suggests that PC accumulation may not be a unique feature of lung fibrosis only in the autoimmune setting. Indeed, IPF lungs showed significantly higher expression of the PC signature (Figure 6C), suggesting that PCs might play a role in pathogenic processes in IPF as well.

### Confirmation of plasma cells in ILD lungs by scRNAseq

**Figure 7:**
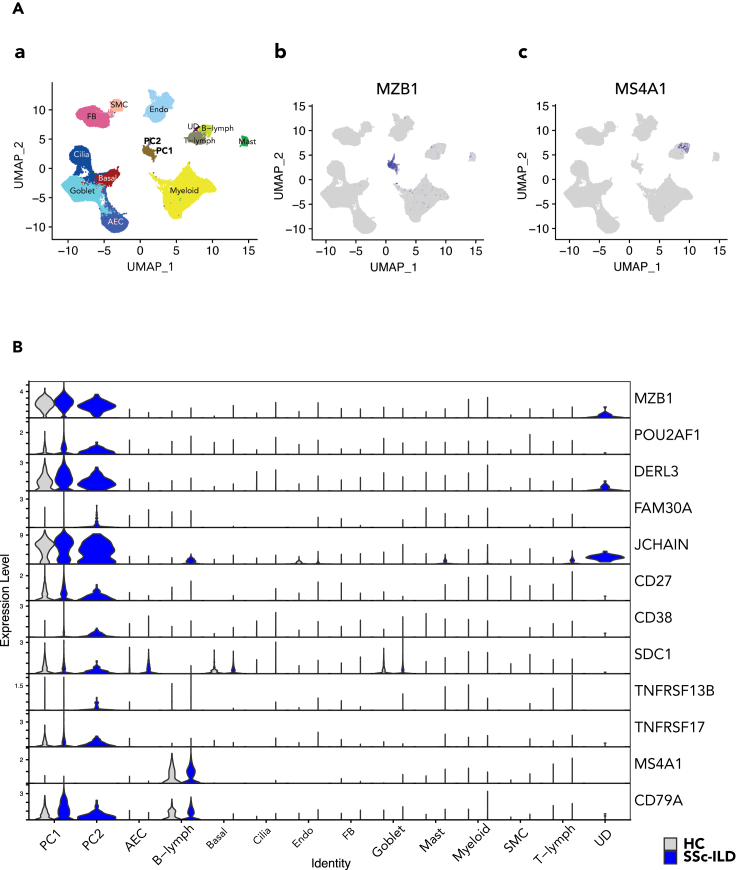
Confirmation of plasma cells in ILD lungs by scRNAseq (A) UMAP atlas of scRNAseq from explanted lung cells depicted an MZB1 expressing subset. The annotated subsets of SSc-ILD lung cells are shown on the left (a) as the reference for cell type position on the UMAP atlas and MZB1 positive (b) and MS4A1 (encoding CD20) (c) cell subsets are depicted with a color spectrum. AEC: alveolar epithelial cells; FB: fibroblasts; SMC: smooth muscle cells; Endo: endothelium; UD: undetermined cells; PC1/PC2, plasma cells. (B) Skin PC signature genes are distinctly expressed in plasma cells in SSc-ILD lung tissue. The gene expression of interest and relevant to B cell development stages are presented in a stacked violin plot in comparison between the control and SSc, where genes related to specific B cell sub-types are listed on the right of y axis and the annotated clusters on x axis. The colors represent the sources of RNAseq, blue from SSc-ILD lung (SSc-ILD), whereas gray from the healthy control (HC). TNFRSF17: encoding BCMA; TNFRSF13B: encoding TACI.

To better delineate the cell population with PC signature and their changes in cellular phenotypes associated with fibrotic diseases, we examined scRNAseq of explanted lungs from ILD patients and controls. scRNAseq analysis showed that small distinct subsets (PC1 and PC2) expressed PC signature genes visualized in single-cell atlas with UMAP plot (Figure 7A) and stacked violin plot (Figure 7B). Their expression levels were higher in SSc-ILD lungs than in control lung (Figure 7B; Figure S7A, Data S3), especially in the PC2 subset which was only detected in SSc-ILD lung (Figure S7B). The clusters of cells also expressed CD38 and CD138 (gene name: SDC1) but were low or negative for CD19 (not shown) and CD20 (gene name: MS4A1) (Figure 7B), which is distinct from conventional CD19 and CD20 positive B lymphocyte cells (Figure 7A). It confirmed that a PC population was present in lung tissue that may contribute to the pathophysiology of ILD, in agreement with a previous report by Schiller et al*.*^22^

## Discussion

Although understanding of the biological processes underpinning the pathogenesis of SSc has improved in recent years due to the vast and continuing progress being made in the domains of genomics, transcriptomics, and proteomics, a gap in their translation into safe, effective, and targeted therapies still exists. A way to narrow that gap would be to evaluate molecular changes in tissue samples and patient data collected in the course of rigorously designed clinical trials for further exploratory research. This would accelerate our understanding about the key molecular pathways operating in affected organs, and how they relate to disease severity, progression, and response (or lack of thereof) to any experimental treatments being studied.

Here, we sought to understand which molecular pathways changed in skin tissue in response to therapeutic blockade of IL-6 signaling in the context of two clinical trials, and how that information could be extrapolated to potentially address the unmet need in SSc. We used available skin punch biopsy samples from individuals enrolled and appropriately consented in the focuSSced cohort as a discovery set, and performed an unbiased whole transcriptome analysis by clustering correlated genes together as modules using the WGCNA approach. This analysis for pharmacodynamic changes showed a PC enriched module that was significantly attenuated in response to TCZ treatment, which we confirmed in samples from the faSScinate trial.

PCs are long-lived tissue-resident antibody secreting cells that generally reside in bone marrow, but are also reported to persist within chronically inflamed tissue such as in kidneys of patients with systemic lupus erythematosus (SLE),^24^ central nervous system of patients with multiple sclerosis,^25^ spleens of patients with auto-immune thrombocytopenia,^26^ inflamed synovial tissue of rheumatoid arthritis patients^27^^,^^28^ or thymus tissue of myasthenia gravis patients.^29^ Long-lived autoreactive PCs implicated in persistent autoimmune inflammation have been reviewed by F. Hiepe et al.^30^ Transcriptomic analyses have not only confirmed the existence of PCs in inflamed tissue but also provided some quantitative relationships with clinical metrics beyond the scope of what is achievable by histopathologic characterization alone. For instance, a transcriptomically derived PC signature was shown to be elevated in the peripheral blood and skin tissue of SSc patients.^31^ While some genes used for the published PC signature are common with our tissue derived PC signature (e.g., JCHAIN), there are differences as well, attributable to the differences in how they were derived. Of note, we did not observe a PC signature in blood that correlated with tissue PC signatures (Figure S5).

Production of pathogenic autoantibodies by self-reactive PCs is a hallmark of autoimmune diseases. We do not have direct data to support that the PCs in the skin tissue are pathogenic or produce autoantibodies. However, although none of the commonly tested systemic autoantibody titers were individually significantly associated with the skin PC signature level, a composite index derived by the sum of the individual autoantibody positivity for each subject, was associated with skin PC level, such that subjects scoring a composite index of two manifested a skin PC signature that was statistically significantly higher than those with a score of one or zero. This suggests that tissue PC score may reflect the totality of the activities of multiple different autoreactive PC (Figure S6). Because the entire spectrum of autoAb specificities in SSc is unknown and not conventionally measurable, a molecular index of total PC activity in tissue as determined by gene expression may provide complementary information to measuring a particular subset of serum autoAb titers. Furthermore, while PCs produce antibodies, they also can produce inflammatory and pro-fibrotic cytokines^32^ which could contribute to tissue pathophysiology independently of antibody-mediated effects. However, this does not explain the disconnect between clinical effects of TCZ treatment on skin and lung outcomes. The lack of available pre- and post-treatment tissue samples from lung precludes a definitive assessment of whether the PD effect on PCs observed in skin is also true in lung tissue. The antibody-independent contributions of PCs to SSc tissue pathology and their roles in ILD represent areas for future investigation.

The putative autoimmune etiology of SSc-ILD is consistent with our observation that a tissue PC signature is elevated and associated with lung function progression. IPF, on the other hand, is associated with compromised regenerative capacity of alveolar epithelium and is not considered an autoimmune disease (though certain auto-antibodies have been reported in some patients^33^^,^^34^^,^^35^^,^^36^), and affects a demographically different population, with a more progressive trajectory. IPF and SSC-ILD, while displaying certain degrees of overlap, are generally differentiated both histopathologically and radiologically (UIP pattern vs. NSIP pattern, respectively). Therefore, our data confirming prior observations that PCs are enriched across multiple fibrotic ILDs despite different pathological patterns imply that dysregulated PC biology may represent a convergent pathological outcome of distinct inciting events. Tissue PCs may contribute to the progression of these apparently disparate ILDs by perpetuating a chronic inflammatory reaction that contributes to persistent tissue injury, fomenting fibrogenesis.

Interleukin-6 (IL-6) is a key regulator of B cell differentiation into PCs and exerts a significant impact on the pathogenesis of PC disorders, including plasmacytoma and myeloma.^37^ Perturbed IL-6 production has been implicated in the development of polyclonal PC abnormalities and PC neoplasias.^38^^,^^39^ Furthermore, IL-6 has been shown to play a crucial role in supporting the generation of long-lived PCs.^40^^,^^41^

Our identified distinct gene signature is enriched for B cell-related genes expressed in various B cell subsets, respectively. While we have referred to this signature as a “PC module,” we acknowledge that it may also reflect B cell activation and the infiltration of activated B cells into SSc tissues. To our knowledge, our analyses demonstrate for the first time that the tissue PC are not only associated with SSc disease at baseline but may also carry prognostic information to help predict clinical progression, and mechanistically link tissue PC levels to endogenous IL-6 activity—a relationship long suspected based on *in vitro* and preclinical observations but not previously demonstrated in human disease via a rigorously controlled, double blind randomized clinical trial setting with a selective IL-6 inhibitor.

Despite the limitations of small sample size from skin biopsies due to the patient consents in focuSSced and the post hoc nature of our study, a plasma cell-associated gene signature measured on a different technical platform in faSScinate yielded similar pharmacodynamic effects. Furthermore, the biological plausibility of our findings is supported by preexisting literature.^26^^,^^30^ Collectively, these features underscore the robustness of the finding, which may provide insight into the contribution of PCs and their regulation by IL-6 in fibrotic diseases. Based on our findings, it is possible that targeting PC numbers and/or function via different mechanisms could represent a viable interventional strategy to ameliorate disease progression in SSc and related fibrotic diseases.

### Limitations of the study

This study has some potential limitations and caveats. The PD effect by Tocilizumab primarily observed in the study is based on the skin biopsies from the clinical trials. The size of samples is limited by the consent of the patients and the original trial design. It is therefore subject to the statistical power and biases in analysis that may have influenced the effect estimates, although the effect has been demonstrated in two independent clinical cohorts in the study. The verification of the plasma gene signature and their change with additional approaches such as IHC imaging which would better support the findings is compromised by the tissue availability since the skin biopsies collected from the clinical trials conducted many years ago have been either lysed to retrieve RNA or destroyed according to the trial protocol by the time we performed the analyses.

Given current techniques to isolate intact PCs from tissues made it challenging to confirm specific tissue B-lineage subsets with the signature, emerging technique such as spatial transcriptomics and proteomics may enable it to be addressed in future studies.

## STAR★Methods

### Key resources table

**Table:** 

REAGENT or RESOURCE	SOURCE	IDENTIFIER
**Deposited data**		

bulk RNAseq of skin biopsy from focuSSced	This paper	GSE231694
Microarray of skin biopsy from faSScinate	Stifano et al.^42^	GSE106358
bulk RNAseq of whole blood PAXgene from faSScinate	This paper	GSE231694
bulk RNAseq of explanted lung tissue from ILD patients	This paper	GSE231694
scRNAseq of fibrotic lungs	Gao et al.^43^	GSE159354

**Software and algorithms**		

R v 4.0.2		http://www.R-project.org
RStudio		http://www.rstudio.com/
DESeq2 package	Bioconductor	https://bioconductor.org/packages/release/bioc/html/DESeq2.html
WGCNA package	CRAN	https://cran.r-project.org/web/packages/WGCNA/index.html
clusterProfiler package	Bioconductor	https://bioconductor.org/packages/release/bioc/html/clusterProfiler.html
GeneOverlap package	Bioconductor	https://bioconductor.org/packages/release/bioc/html/GeneOverlap.html
Seurat package	CRAN	https://cran.r-project.org/web/packages/Seurat/index.html

### Resource availability

#### Lead contact

Further information and requests for resources and reagents should be directed to and will be fulfilled by the lead contact, Guiquan Jia (jjia@gene.com).

#### Materials availability

This study did not generate new unique reagents.

### Method details

#### RNAseq dataset

The RNA for bulk RNAseq was prepared from the skin biopsies collected from focuSSced cohort (NCT02453256). Briefly, the skin samples in RNAlater (Qiagen, Austin, TX, USA) were transferred to TRIzol (ThermoFisher Scientific, Waltham, MA, USA)-filled tubes and homogenized using a Miltenyi gentleMACS™ Dissociator. Afterwards, samples were centrifuged for 3 minutes at 8000g and the supernatants were collected. The total RNA was isolated from the supernatant using the RNeasy Fibrous Tissue mini kit. Resultant total RNA after assessed quantitatively (NanoDrop; ThermoFisher Scientific) and qualitatively (Bioanalyzer; Agilent, Santa Clara, CA, USA) were used for RNAseq.

Bulk RNAseq dataset was prepared by sequencing the RNA on the Illumina HiSeq 4000 platform and followed by aligned and annotated to human genomic database GRCh38, which comprised 58302 features and 76 samples, 62 from 37 subjects, the subset of the patients that consented for pre-post treatment skin biopsies in focuSSced study and 14 from age and gender matched healthy donors. The statistical diagnostic for flagging potential outlying samples was analyzed with the standard procedure of WGCNA^44^^,^^45^ using all genes available. Three samples as the outliers were removed from the subset of SSc patients in this dataset. Among 36 subjects with SSc after outlier sample removal, 22 subjects had 2 biopsies, each from pre- (baseline) and post-treatment (week 48), where 11 subjects were in placebo (PBO) arm and 11 subjects in the TCZ treatment arm.

Microarray dataset of skin biopsy from faSScinate cohort (NCT01532869) was described in previous publication.^14^^,^^42^

Bulk RNAseq dataset of whole blood PAXgene generated in Genentech included 20 samples from patients in faSScinate (NCT01532869) and 10 age matched healthy donors and was aligned and annotated to the genome database GRCh38.

Bulk RNAseq dataset of explanted lung tissue from ILD patients was generated in Genentech using lung samples collected from SSc ILD and IPF patients during transplantation. It included 20 SSc ILD, 20 IPF and 20 healthy controls. It was aligned and annotated to the genome database GRCh38.

scRNAseq dataset was described in previous publications,^43^ which included cells of lung tissues from 3 patients with systemic sclerosis and 3 normal controls (GSE159354).

#### Identification of DEGs in SSc skin and ILD lung in compared with the controls

DESeq2 package^46^ in R was applied to identify DEGs at baseline by comparing expression counts of baseline samples between disease and control. The adjusted p-value < 0.05, |fold change (FC)| ≥ 2, and mean of counts (baseMean, a parameter used in DEseq2) > 25 (about one quartile of the range of mean of counts for genes in RNAseq) were used as cut-off criteria for DEGs selection for further analysis indicated (detail of DEG information available in supplemental information).

#### Identification of DEGs by the effect of TCZ treatment in SSc skin

DEG analysis with DESeq2 package was performed with likelihood ratio test (LRT) in a matrix design of time course analysis with fully-paired samples intra-individually to reach the differential expression (DE) between pre- (baseline, used as the reference) and post-treatment (week 48) within each arm and the gene expression impacted by the true effect of TCZ treatment was obtained by contrasting between two arms with placebo group as the reference. The PD genes were selected based on the criteria as of adjusted p-value < 0.05, FC ≤ -1.5, and mean of counts (baseMean) > 25 (about one quartile of range of mean of counts for genes in RNAseq). The information of PD genes is available in supplemental information.

#### Construction of the gene co-expression network affected by TCZ treatment

All PD genes selected on the effect of TCZ treatment and the subset of RNAseq from SSc skin samples in the treatment arm were used in the construction of weighted co-expression networks (modules) using the WGCNA^44^^,^^47^ procedure with WGCNA R package available on the Comprehensive R Archive Network (http://cran.r-project.org). Briefly, the gene expression level in counts were transformed with the function of voom in limma^48^ R package first. And then the network was rendered based on a pair-wise correlation using a β of 8 as a weight and 20% genes in the least connection from softConnectivity analysis within the package were excluded from the clustering. And finally co-expressed genes were clustered according to a topological overlap metric with the method of “ward.D2”, and the modules assigned in default by colors with the dynamic tree cut algorithm at a minimum module size of 30 for gene number. The genes without annotated gene symbol were excluded (95 out of 427 genes). Module eigengene (ME) was calculated with a function in WGCNA package based on all gene expression within the module/signature and transformed to positive value with formula (ME – min (ME) +1) when required in the process of log transform.

#### Construction of the gene co-expression network in ILD lungs

All DEGs from DEG analysis described in relevant section were applied to construct weighted co-expression modules for each case. The subset of RNAseq from the defined disease samples were used in the network construction using the same parameter setups in the procedure of WGCNA described above.

#### Pathway enrichment analysis

In order to investigate gene module at the molecular and functional level, we performed Reactome pathways enrichment analysis (and other pathway databases if available) by using clusterProfiler^49^ (http://bioconductor.org/packages/release/bioc/html/clusterProfiler.html), which calculates and compares enriched functional categories of each gene module.^50^ Using gene ratios (the proportions of genes enriched in each category) and adjusted p-values, a dot plot was constructed to visualize the difference in enriched functional categories between the modules.

#### Gene overlap analysis for the conserved PC module

The principle to detect module overlap network was elicited previously by Mohoney et al.^51^ and Taroni et al.^23^ As an alternative practice, R Package: GeneOverlap from BioConductor, was used. Given two sets of gene lists, this package calculated the overlaps between all pairs of lists from the two intersecting sets. Fisher's exact test was used based on the hypergeometric distribution to determine the p-value and odds ratio in comparison to a genomic background. The null hypothesis is that the odds ratio is no larger than 1. In our cases, the genomic background was scoped as the union size of all DEG input for the sets in comparison presented in the result section, respectively. The matrix table with odd ratio and p value were created to represent all pairwise overlap comparisons between the gene lists, where p value was transformed with -log_10_(x). The p value less than 0.05 was labelled as no significance (n.s.) statistically.

#### Heatmap of genes in clustering

A common method of visualizing gene expression as a heatmap was generated with pheatmap function from R package of pheatmap available from CRAN. All gene expression in counts from RNAseq was voom-transformed with limma^48^ package for linear modeling and scaled in the heatmap. The genes and subjects in raw and column of heatmap, respectively, were hierarchically clustered with the method of ward.D2.

#### scRNAseq analysis

The scRNAseq data was processed and analyzed following the standard analytical flow on the website of Seurat^52^ R package version 4.0.1. In brief, after counts normalization and IntegrationAnchors identification for each sample dataset, respectively, all sample datasets were integrated together with the functions in Seurat package accordingly. Then we performed graph-based unsupervised clustering of this integrated data using FindNeighbors (reduction = “pca”, dims = 1:30) and FindClusters (resolution = 1) function of Seurat and visualized the clusters on uniform manifold approximation and projection (UMAP) plot using RunUMAP function of Seurat based on networks of the shared-nearest neighbor (SNN) graph. Cell types for each cluster were annotated via canonical cell type markers and then merged to the major cell types presented in airway and lung. The expression comparison of the gene of interest was plotted in stacked violin plot with the clusters of interest using the functions included in Seurat package.

Boxplots representing the nonzero percent makeup distributions of individual varieties of immune cell as a proportion of all immune cells per subject within each disease group.

### Quantification and statistical analysis

All statistical analyses and graphing were performed using R with a variety of relevant packages available either form CRAN or BioConductor. Statistically correlation coefficient and significant differences were assessed by the tests indicated in the graphs. For all analyses, a two-sided p-value less than 0.05 was considered statistically significant.

### Additional resources

focuSSced cohort: NCT02453256. Link: https://clinicaltrials.gov/study/NCT02453256

faSScinate cohort: NCT01532869. Link: https://clinicaltrials.gov/study/NCT01532869.

## Data Availability

• All RNA-seq data have been deposited at GEO and are publicly available as of the date of publication. Accession numbers are listed in the key resources table. • This paper does not report original code. • Any additional information required to reanalyze the data reported in this paper is available as supplemental information or from the lead contact upon request.

## Supplemental information

Document S1. Figures S1–S7

Data S1. DEG list, related to Figure 2

Data S2. Module gene list, related to Figure 2

Data S3. DEG in scRNAseq, related to Figure 7
