# Supplementary material for: An interleukin 6 responsive plasma cell signature is associated with disease progression in systemic sclerosis interstitial lung disease
Source: iScience. 2023 Oct 5;26(11):108133. doi: 10.1016/j.isci.2023.108133 (PMC10585397; doi:10.1016/j.isci.2023.108133)
Supplement: Document S1. Figures S1–S7 [file mmc1.pdf]

iScience, Volume *26*

Supplemental information

An interleukin 6 responsive plasma cell signature is associated with disease progression in systemic sclerosis interstitial lung disease

Guiquan Jia, Thirumalai R. Ramalingam, Jason Vander Heiden, Xia Gao, Daryle DePianto, Katrina B. Morshead, Zora Modrusan, Nandhini Ramamoorthi, Paul Wolters, Celia Lin, Dinesh Khanna, and Joseph R. Arron

Supplementary Materials

Fig.S1. Subjects with higher baseline PC module expression showed greater PD effect within TCZ treatment group, whereas such a trend was not apparent in the placebo group, related to Figure 2D.

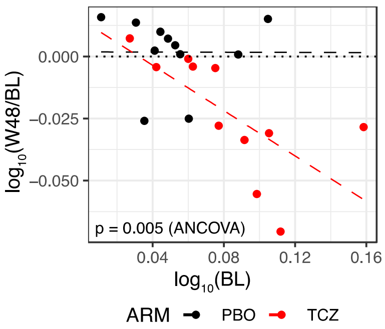

The scatterplot shows the function of change (follow-up / baseline measurements) against baseline measurements, both in log10. The dotted line represents perfect agreement (no change) and the dashed lines are fitted regression lines for TCZ treatment and placebo groups, respectively. P-value was from ANCOVA test.

Fig.S2. High correlation of 4-gene eigengene with 44-gene eigengene, related to Figure 2.

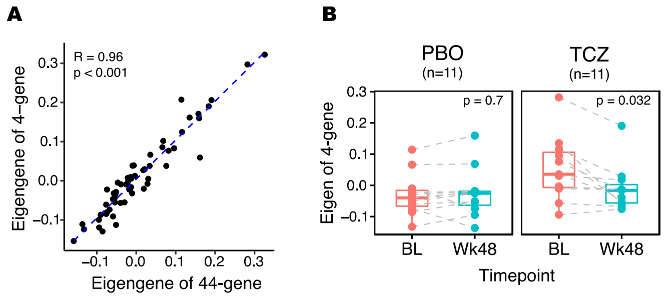

Fig.S2A. High correlation of 4-gene eigengene with whole PC module (44-gene) within SSc patients. The eigengene of 4-gene (MZB1, POU2AF, DERL3 and FAM30A) were calculated

and compared with that of whole PC module in the scatterplot. The Pearson correlation coefficient and p value are shown in the legend.

Fig.S2B. Paired comparison of module eigengene of 44-genes at baseline and 48 weeks following-up. Within-patient comparison showed a same significant reduction of ME of 44-genes in patients with TCZ treatment but not with placebo (PBO) as the ME of 4-genes in Fig.2D.

Fig.S3. High pair-wise correlation of PC module genes in FaSScinate, related to Figure 3A.

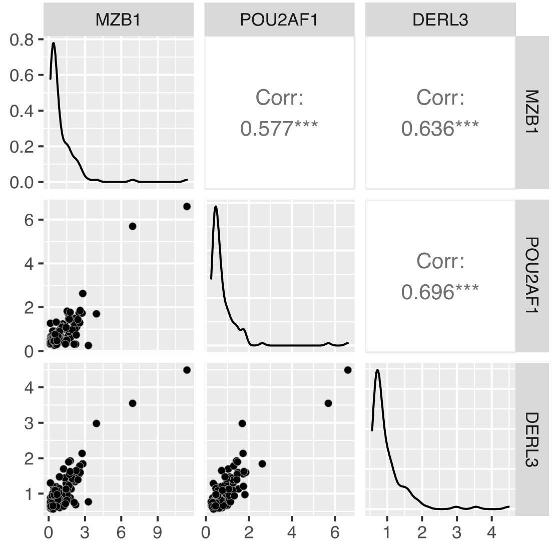

The plot shows the scatterplots for all possible pairs of 3 genes of PC module used for PD effect of the TCZ treatment, combining with the result of correlation coefficients and spearman correlation tests (the asterisks next to the coefficients). The axis on the left and the bottom represent the gene expression level from microarray, corresponding to each gene labelled on the right and the top, respectively. *** : p-value < 0.001.

Fig.S4. Not high association of baseline PC ME with other clinical traits (Baseline FVC, MRSS, DLCO) was observed in FocuSSced cohort, related to Figure 4.

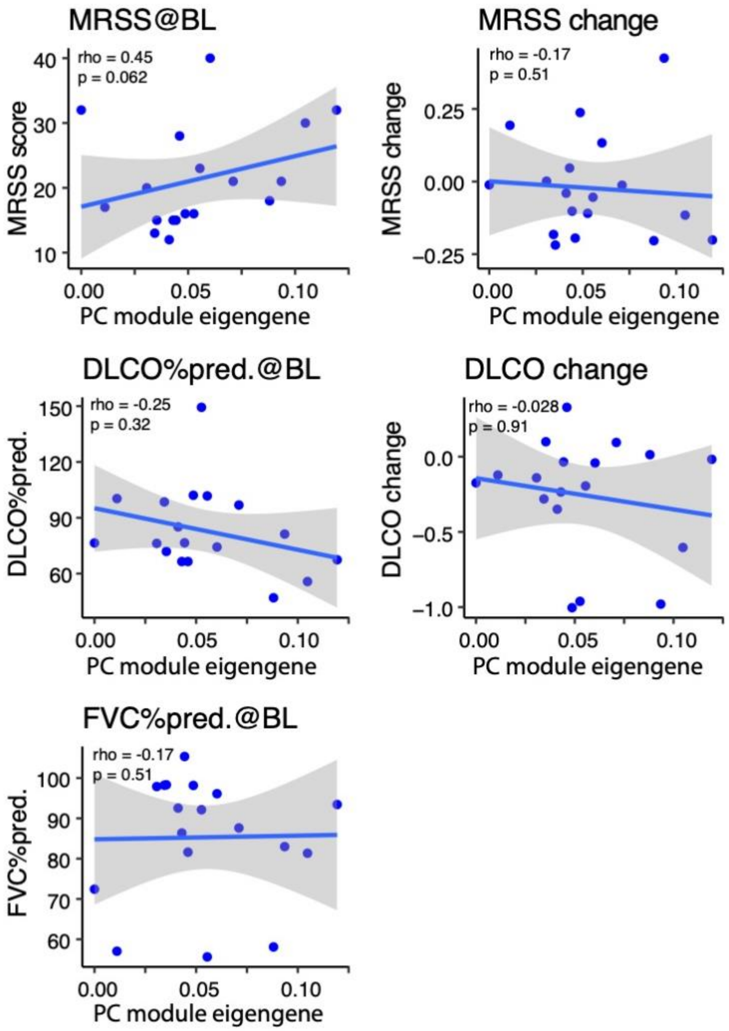

Fig.S5. The heatmap showed PC signature genes neither highly correlated nor all elevated in peripheral blood from SSc-ILD in faSScinate cohort compared to controls, related to Figure 5.

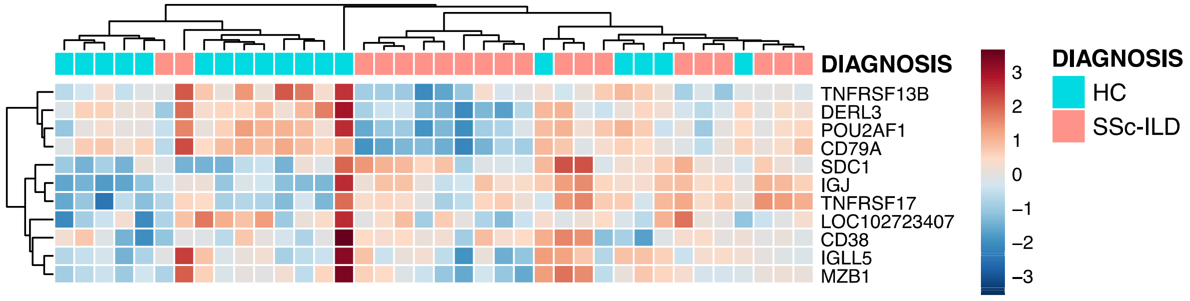

The genes in PC signature with other B and plasma cell relevant genes (CD79A, IGJ and TNFRSF13B) presented in rows and subjects in columns from RNAseq of PAXgene from FaSScinate cohort were hierarchically clustered and gene expression was transformed and standardized to z-score. The conventional PC marker CD138 (encoded by SDC1) was included as a general reference.

Fig.S6. **The relationship between the skin PC signature with common SSc-related autoantibodies, related t to the discussion.**

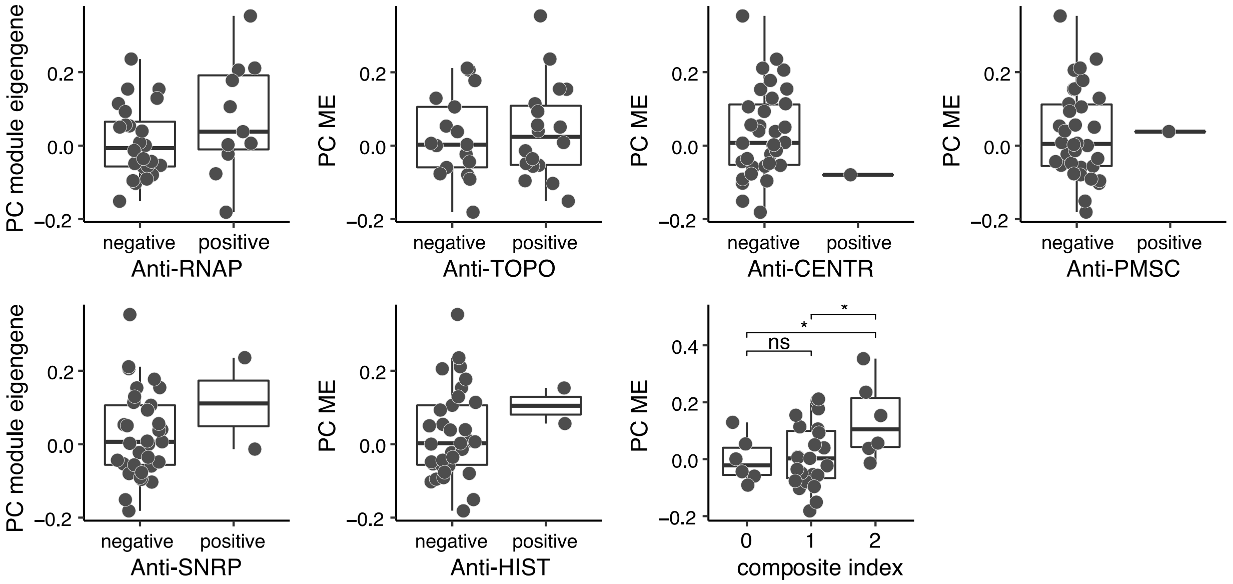

Six types of auto-Abs were tested at baseline for positive in SSc patients from FocuSSed cohort. The composite index is the sum of positives from 6 individual tests (0: no positive of 6 tests; 1: one positive of either; 2: two positives). * p-value < 0.05 by Wilcoxon test. Anti-RNAP: Anti RNA Polymerase Antibody; anti-TOPO: Anti-Topoisomerase Ab; Anti-CENTR: Anti-Centromere Ab; Anti-PMSC: Anti PM/SCL Ab; Anti-SNRP: Anti U1 Sm Nuc Ribonucleoprot Ab; Anti-HIST: Anti Histone Ab.

Fig.S7. The comparison of PC subclusters from scRNAseq analysis between SSc-ILD lung and HC lung, related to Figure 7.

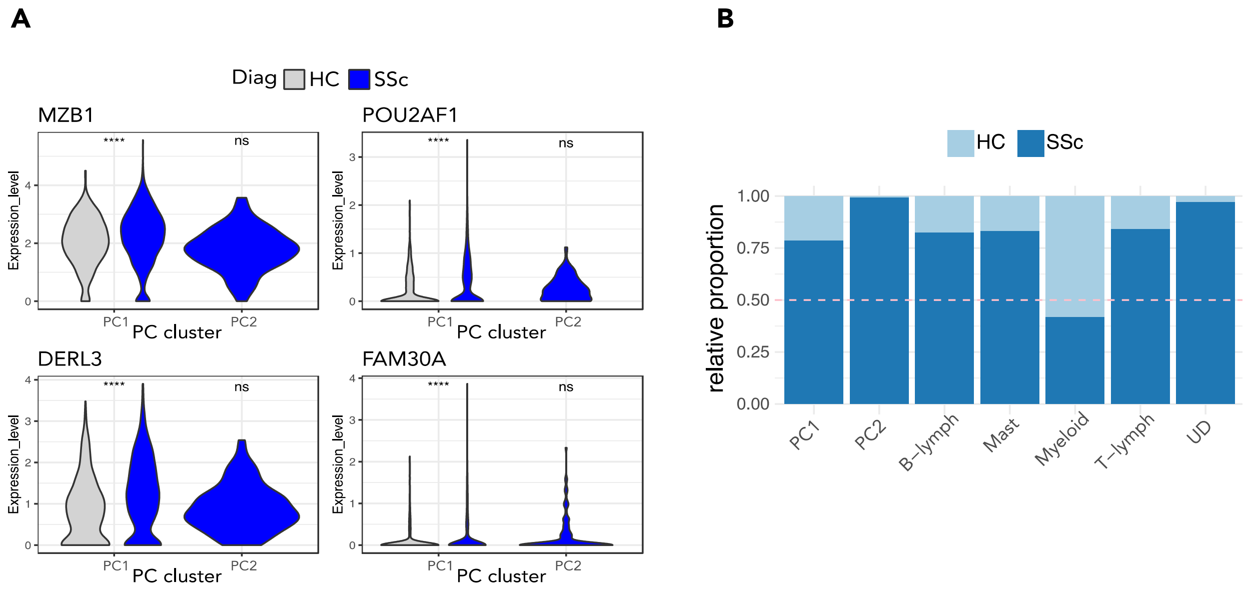

Fig.S7A. The comparison of PC gene expression between SSc-ILD and HC

The expression of 4 PC genes from scRNAseq is presented in a violin plot in comparison between SSc and the control. The statistical significance on the adjusted p-value is labelled on the top of the graph for each PC cluster, defined as the following: **** = significant less than 0.001 level, based on the statistic test, MAST which is used to identify differentially expressed genes between two groups of cells using a hurdle model tailored to scRNA-seq data.

Fig.S7B. The comparison of proportion of PC frequency in CD45+ enriched population between SSc-ILD and HC

Bar plot showing the fraction of each cell type according to the origin of samples
